# Methylation regulation of Antiviral host factors, Interferon Stimulated Genes (ISGs) and T-cell responses associated with natural HIV control

**DOI:** 10.1371/journal.ppat.1008678

**Published:** 2020-08-06

**Authors:** Bruna Oriol-Tordera, Maria Berdasco, Anuska Llano, Beatriz Mothe, Cristina Gálvez, Javier Martinez-Picado, Jorge Carrillo, Julià Blanco, Clara Duran-Castells, Carmela Ganoza, Jorge Sanchez, Bonaventura Clotet, Maria Luz Calle, Alex Sánchez-Pla, Manel Esteller, Christian Brander, Marta Ruiz-Riol

**Affiliations:** 1 IrsiCaixa AIDS Research Institute, Hospital Germans Trias i Pujol, Institute for Health Science Research Germans Trias i Pujol (IGTP), Badalona, Spain; 2 Cancer Epigenetics and Biology Program (PEBC), Bellvitge Biomedical Research Institute, L'Hospitalet de Llobregat, Barcelona, Spain; 3 Josep Carreras Leukaemia Research Institute (IJC), Badalona, Spain; 4 University of Vic—Central University of Catalonia, Catalonia, Vic, Spain; 5 Fundació Lluita contra la Sida, Infectious Disease Department, Hospital Universitari Germans Trias i Pujol, Badalona, Spain; 6 Catalan Institution for Research and Advanced Studies (ICREA), Barcelona, Spain; 7 Asociación Civil IMPACTA Salud y Educacion, Lima, Peru; 8 Alberto Hurtado School of Medicine, Universidad Peruana Cayetano Heredia, Lima, Peru; 9 Department of Global Health, University of Washington, Seattle, Washington, United States of America; 10 Centro de Investigaciones Tecnológicas, Biomédicas y Medioambientales, CITBM, Lima, Peru; 11 Statistics Department, Biology Faculty, University of Barcelona, Spain; 12 Statistics and Bioinformatics Unit Vall d'Hebron Institut de Recerca (VHIR), Spain; 13 Centro de Investigacion Biomedica en Red Cancer (CIBERONC), Madrid, Spain; 14 Physiological Sciences Department, School of Medicine and Health Sciences, University of Barcelona (UB), Barcelona, Catalonia, Spain; 15 AELIX Therapeutics, Barcelona, Spain; Vaccine Research Center, UNITED STATES

## Abstract

GWAS, immune analyses and biomarker screenings have identified host factors associated with *in vivo* HIV-1 control. However, there is a gap in the knowledge about the mechanisms that regulate the expression of such host factors. Here, we aimed to assess DNA methylation impact on host genome in natural HIV-1 control. To this end, whole DNA methylome in 70 untreated HIV-1 infected individuals with either high (>50,000 HIV-1-RNA copies/ml, n = 29) or low (<10,000 HIV-1-RNA copies/ml, n = 41) plasma viral load (pVL) levels were compared and identified 2,649 differentially methylated positions (DMPs). Of these, a classification random forest model selected 55 DMPs that correlated with virologic (pVL and proviral levels) and HIV-1 specific adaptive immunity parameters (IFNg-T cell responses and neutralizing antibodies capacity). Then, cluster and functional analyses identified two DMP clusters: cluster 1 contained hypo-methylated genes involved in antiviral and interferon response (e.g. *PARP9*, *MX1*, and *USP18*) in individuals with high viral loads while in cluster 2, genes related to T follicular helper cell (Tfh) commitment (e.g. *CXCR5* and *TCF7*) were hyper-methylated in the same group of individuals with uncontrolled infection. For selected genes, mRNA levels negatively correlated with DNA methylation, confirming an epigenetic regulation of gene expression. Further, these gene expression signatures were also confirmed in early and chronic stages of infection, including untreated, cART treated and elite controllers HIV-1 infected individuals (n = 37). These data provide the first evidence that host genes critically involved in immune control of the virus are under methylation regulation in HIV-1 infection. These insights may offer new opportunities to identify novel mechanisms of *in vivo* virus control and may prove crucial for the development of future therapeutic interventions aimed at HIV-1 cure.

## Author summary

The infection with the human immunodeficiency virus (HIV), as for other viral infections, induce global DNA Methylation changes in the host genome. Herein, we identified for first time the methylation impact on host genome in untreated HIV-1 infection with different degrees of *in vivo* virus control. Specifically, we observed that individuals with a better HIV-1 control showed a hypermethylation of genes associated with antiviral and interferon pathways and the hypomethylation of genes associated with the differentiation process of T follicular helper cells. Interestingly, these epigenetic imprints in host genome were strongly correlated with virus content and HIV-specific T cell responses. Therefore, we propose DNA Methylation as the regulation mechanism of host genes involved in immune HIV-1 control that could interfere in the efficacy of cure strategies. We also highlight the importance of DNA Methylation to regulate immune responses not only in HIV-1 but also in chronic infections or other pathologic situations associated with a sustained activation of the immune system.

## Introduction

Treatment of HIV-1 infected individuals with antiretroviral drugs (combination antiretroviral therapy, cART) is highly effective in suppressing viral replication to undetectable levels. However, the virus persists in the body in form of a proviral reservoir in latently infected cells and cART needs therefore to be taken for life. Given that the access and adherence to the treatment might be limited, especially in low-income countries and considering the health consequences of long-term cART use, stigma and high costs, there is a need for the development of new strategies to achieve a so-called functional cure and/or interventions that can eliminate the virus from the body [1]. The study of the small-subset of HIV-1 controllers, i.e. HIV-1-infected people that can naturally maintain undetectable or low HIV-1 plasma viral load (pVL) without cART, have been of relevance to understand the mechanisms of spontaneous HIV-1 viral control [2]. Such control has been mainly linked to host factors, especially HLA genetics and antiviral immunity mediated by HIV-1-specific CD8 and CD4 T-cells, as well as to virological factors including viral mutations that reduce viral replicative fitness [3]. Despite this progress, there remain significant gaps in the understanding of spontaneous virus control and how the maintenance of HIV-1 latency can be broken [4,5].

For several viral infections, different epigenetic changes have been described in the genomes of both, the virus and the host [6]. Some of these epigenetic changes, in particular DNA methylation of genes, can be induced upon initial infection. The process is driven mainly by the increase of DNA Methyltransferases (DNMT), the enzymes that catalyze the transfer of methyl groups to cytosine residues of DNA. At the level of viral DNA, several reports have suggested that the hypermethylation of 5’LTR regions of HIV-1 proviral is of relevance for latency maintenance [7]. In contrast, for methylation changes of host genes, most reports to date have assessed methylation levels of single host genes and only a few studies exist that determined DNA methylation of whole host genomes in HIV-1 infection [8–10]. Among these, the analysis of PBMC DNA methylation patterns in a pair of monozygotic twins sero-discordant for HIV-1 infection, revealed an overall increase in host DNA methylation in the HIV-1 infected compared to the HIV-1 uninfected twin [10]. A subsequent study that assessed differential DNA methylation patterns between HIV-1 infected and uninfected individuals, identified altered epigenetic regulation of host factors such as *NLRC5* and *LPCAT1* [9]. These studies indicate that HIV-1 infection may leave marked changes in the host DNA methylation patterns that could affect the expression of host factors involved in viral replication as well as in innate and adaptive immune defense. In light of ongoing efforts to restore effective antiviral immunity and achieve sustained functional cure of HIV-1 infection, it will be critical to i) identify such methylation-dependent epigenetic imprints in HIV-1 infection, ii) understand their effects on antiviral immunity and importantly, iii) find ways to not only transiently restore antiviral immune responses but to correct their underlying epigenetic dysregulation to achieve lasting cure [11].

In this report, full host-genome DNA methylation analysis in a cohort of HIV-1 infected individuals with variable ability to spontaneously control viral replication *in vivo*, reveal for the first time, key methylation signatures in peripheral blood predictive of HIV-1 plasma viral loads and proviral levels, as well as HIV-1-specific adaptive immunity, associated with natural HIV-1 control. Therefore, these data strongly support an important role of epigenetics as a regulation mechanism behind HIV-1 control that should be considered in future therapeutic strategies.

## Results

### Differential DNA methylation patterns in PBMC of untreated HIV-1 chronically infected individuals with variable levels of HIV-1 pVL

With the aim of determining DNA methylation patterns that could be predictive of viral control, PBMC isolated from 70 HIV-1 infected individuals were subjected to a genome-wide DNA methylation analysis. This cohort included a group of subjects with high (HIV-High; pVL >50,000 HIV-1 RNA copies/ml, n = 29) or low HIV-1 viral loads (HIV-Low; pVL <10,000 HIV-1 RNA copies/ml, n = 41).

**Fig 1:**
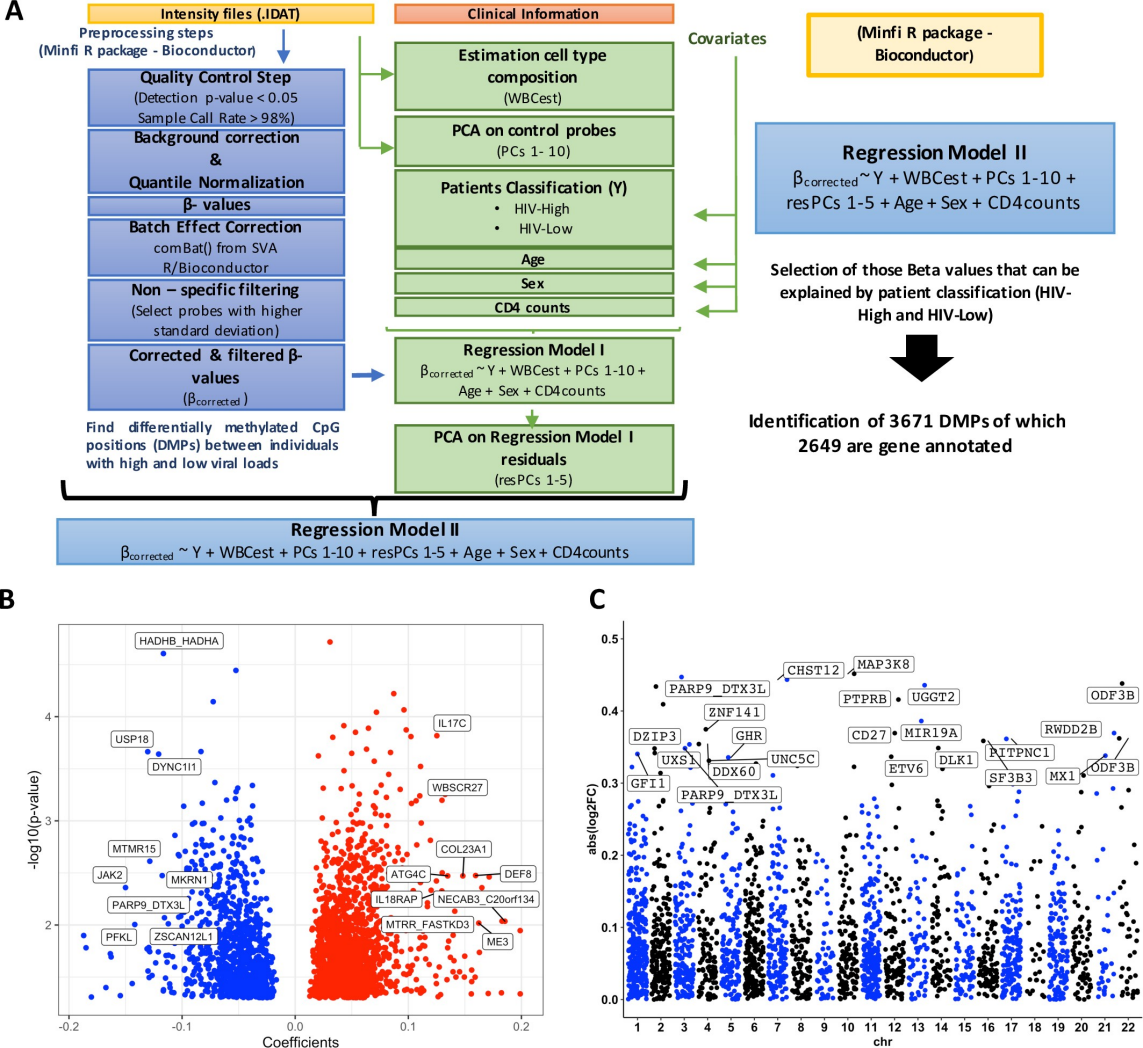
Differentially methylated CpG positions between HIV-1 infected patients with different ranges of plasma viral loads. (A) Analysis strategy for the identification of differentially methylated positions (DMPs). (B) Volcano Plot showing 2,649 gene annotated DMPs (p-value is shown in Y-axis and the regression coefficient from de model on the X-axis). Blue indicates hypomethylation and red, hypermethylation in HIV-High group. Labels indicate top 9 CpG sites with a coefficient higher than |0.1| in HIV-High or HIV-Low. (C) The Manhattan Plot shows the identified DMPs, according the log2 Fold-Change (FC) (Y-axis) and their location on different chromosomes (X-axis).

After a pre-processing step (Fig 1A), 56,513 CpG positions were left to be analyzed for methylation differences between the two groups. A previously described linear model-based approach that adjusts for methylation confounders (including sex, age and estimated cell type proportion [9,12]) was combined with a correction for individuals´ CD4 counts due to the significant differences between both groups for this parameter. This analysis identified a total of 3,671 differentially methylated positions (DMPs) considering a p-value < 0.05 as cut-off (Fig 1A). Only the DMPs between both groups of HIV-1 infected individuals that had a gene annotation (n = 2,649) were kept for further analyses. Of them, n = 1,668 (63%) were found in CpG Islands (CGIs) regions: including 29% in the island itself, 27.5% in CpG shores (2Kb from CGI), and 6.5% in CpG shelves (4 Kb from CGI). A total of n = 1,536 (58%) of DMP were localized in promoter regions of the genes (including TSS1500, TSS200, 5’UTR and 1^st^ Exon regions), suggestive of a potential effect on gene expression levels [13]. Fig 1B shows 224 CpG positions hypomethylated (blue) and 910, hypermethylated (red) in HIV-High individuals. Overall, DMPs were widely distributed across the whole genome, with chromosomes 3, 11, 12 and 16 containing the highest number of DMPs relative to the number of analyzed CpG positions per chromosome (Chi-squared test, p < 0.05, Fig 1C). These data highlight that there exist distinct DNA methylation patterns widely distributed across the entire host genome that differentiate chronically HIV-1 infected individuals with spontaneous high or low pVL.

### Relative control of *in vivo* HIV-1 replication is associated with DMPs in gene encoding proteins involved in adaptive and innate immune responses

To select differentially methylated positions (DMPs) that could discriminate between individuals with high or low HIV-1 pVL and to be used in downstream analyses, we applied a random forest model with 1000 iterations and 5-fold cross-validations [14,15] on the 2,649 gene annotated DMPs (AUC = 0.92). This resulted in the identification of 55 DMPs, of which 26 (47%) were located in CpG islands of promoter regions (Table 1). The most selected DMP, cg22930808, is located in the promoter of *PARP9-DTX3L* gene on chromosome 3 (hypermethylated in HIV-Low, Table 1).

**Table 1:** Classificatory CpG positions into the groups of HIV-High or HIV-Low.

CpG position	*Gene*	Chr	Relation to Island	Relation to Gene	Mean Beta-value (HIV-Low)	Mean Beta-value (HIV-High)	p-value ^A^	Frequency Random Forest ^B^	Cluster	Expected Expression ^c^
cg22930808	*PARP9_DTX3L*	chr3	N_Shore	5UTR_TSS1500	0.76	0.56	0.02	821	1	↓
cg20098015	*ODF3B*	chr22	S_Shore	TSS200	0.64	0.5	0.05	724	1	↓
cg01028142	*CMPK2*	chr2	N_Shore	Body	0.88	0.8	<0.01	643	1	-
cg23221113	*ODF3B*	chr22	S_Shore	5UTR_1stExon	0.29	0.22	0.02	613	1	↓
cg20939114	*PITPNC1*	chr17	OpenSea	Body	0.26	0.33	0.02	607	2	-
cg10152449	*CHST12*	chr7	S_Shore	5UTR	0.56	0.41	0.05	458	1	↓
cg02217713	*PRKAR1B*	chr7	N_Shore	Body	0.71	0.62	0.05	452	1	-
cg08122652	*PARP9_DTX3L*	chr3	N_Shore	5UTR_TSS1500	0.77	0.6	0.02	430	1	↓
cg02538772	*SND1*	chr7	OpenSea	Body	0.57	0.64	0.04	374	2	↑
cg15413523	*TCF7*	chr5	S_Shore	5UTR_1stExon	0.47	0.54	0.04	308	2	↑
cg20651018	*CARS*	chr11	OpenSea	Body	0.52	0.64	0.02	308	2	-
cg14392283	*LY6E*	chr8	N_Shelf	3UTR	0.84	0.76	0.02	301	1	-
cg13015616	*HFE*	chr6	OpenSea	TSS1500	0.61	0.71	0.03	264	2	↑
cg26005232	*NUDC*	chr1	N_Shore	TSS1500	0.48	0.56	0.04	240	2	↑
cg11224765	*ODF3B*	chr22	S_Shore	TSS200	0.58	0.48	0.01	231	1	↓
cg08863939	*TLK1*	chr2	OpenSea	5UTR	0.46	0.55	0.04	225	2	↑
cg08926253	*IRF7*	chr11	Island	Body	0.74	0.66	0.01	225	1	-
cg21535657	*ETV6*	chr12	S_Shelf	Body	0.32	0.25	0.01	168	1	-
cg04537602	*CXCR5*	chr11	OpenSea	Body_TSS1500	0.53	0.63	0.05	154	2	↑
cg02679745	*C9orf139_FUT7*	chr9	S_Shore	Body_TSS1500	0.64	0.56	0.03	148	1	↓
cg11477010	*ANKFY1*	chr17	OpenSea	Body	0.67	0.73	0.01	141	2	-
cg01190666	*PRIC285*	chr20	N_Shore	5UTR	0.72	0.66	0.02	126	1	↓
cg22862003	*MX1*	chr21	N_Shore	TSS1500_5UTR	0.75	0.62	0.05	124	1	↓
cg06981309	*PLSCR1*	chr3	N_Shore	5UTR	0.75	0.62	0.02	107	1	↓
cg02297838	*MIR19A_MIR17HG*	chr13	S_Shore	TSS1500_Body	0.52	0.4	0.02	106	1	↓
cg01774027	*ARID3A*	chr19	Island	Body	0.6	0.52	0.02	95	1	-
cg20631044	*C19orf54_SNRPA*	chr19	N_Shore	TSS200_TSS1500	0.68	0.61	0.03	95	1	↓
cg18338046	*TCF7*	chr5	S_Shore	Body	0.58	0.69	<0.01	90	2	-
cg01623438	*CTSZ*	chr20	S_Shore	TSS1500	0.66	0.6	0.01	89	1	↓
cg14293575	*USP18*	chr22	S_Shelf	5UTR	0.72	0.6	0.03	78	1	↓
cg06164260	*BCL6*	chr3	N_Shore	5UTR_TSS200	0.61	0.55	0.01	76	1	↓
cg19400179	*DZIP3*	chr3	OpenSea	5UTR	0.37	0.47	0.03	70	2	↑
cg02676052	*LCP2*	chr5	OpenSea	TSS1500	0.62	0.67	0.02	69	2	↑
cg14191134	*LOC283050*	chr10	S_Shore	Body	0.37	0.3	0.05	68	1	-
cg21549285	*MX1*	chr21	S_Shore	5UTR	0.77	0.61	0.01	67	1	↓
cg00808969	*USP35_KCTD21*	chr11	N_Shore	TSS1500_5UTR	0.41	0.48	0.01	66	2	↑
cg13298528	*CXCR5*	chr11	OpenSea	Body_TSS1500	0.57	0.67	0.04	60	2	↑
cg15065340	*TNK2*	chr3	N_Shelf	5UTR	0.7	0.62	0.03	58	1	↓
cg05883128	*DDX60*	chr4	N_Shore	5UTR	0.48	0.37	0.04	45	1	↓
cg19368911	*KIF26B*	chr1	OpenSea	Body	0.5	0.58	0.05	45	2	-
cg00688810	*SHBG_FXR2*	chr17	N_Shore	TSS1500_Body	0.72	0.78	0.04	43	2	↑
cg03043696	*GNB1*	chr1	N_Shore	5UTR	0.61	0.55	0	43	1	↓
cg10435235	*ARHGEF7*	chr13	OpenSea	3UTR	0.74	0.69	0.03	42	1	-
cg27519392	*CHD7*	chr8	OpenSea	Body	0.63	0.71	0.03	40	2	-
cg26567688	*COL4A3BP*	chr5	OpenSea	Body	0.67	0.74	0.05	29	2	-
cg08179431	*HFE*	chr6	OpenSea	TSS1500	0.7	0.79	0.03	27	2	↑
cg12042587	*GHR*	chr5	N_Shore	TSS200	0.22	0.28	0.01	27	2	↑
cg22036538	*CD27_LOC678655*	chr12	OpenSea	1stExon_5UTR	0.34	0.44	0.02	27	2	↑
cg20321801	*CD81*	chr11	N_Shelf	Body	0.59	0.53	0.01	27	1	-
cg04858110	*SCRN1*	chr7	OpenSea	5UTR_Body	0.66	0.72	0.04	24	2	↑
cg12217560	*TNFRSF19*	chr13	OpenSea	1stExon_5UTR	0.51	0.58	0.04	21	2	↑
cg06872964	*IFI44L*	chr1	OpenSea	TSS1500	0.7	0.59	0.02	19	1	↓
cg07892167	R3HCC1	chr8	N_Shore	TSS1500	0.52	0.45	0.04	17	1	↓
cg14659511	DOCK9	chr13	OpenSea	Body	0.62	0.69	0.01	14	2	-
cg02385820	RINL	chr19	N_Shore	3UTR	0.71	0.67	<0.01	13	1	-

^A^ p-value of the regression model applied to determine DMPs.

^B^ CpG positions ordered according the frequency of selection by random forest model.

^C^ For CpGs in promoters, it is shown the expected gene expression in case of an epigenetic regulation. Values are relative to HIV-Low group.

Chr = Chromosome.

**Fig 2:**
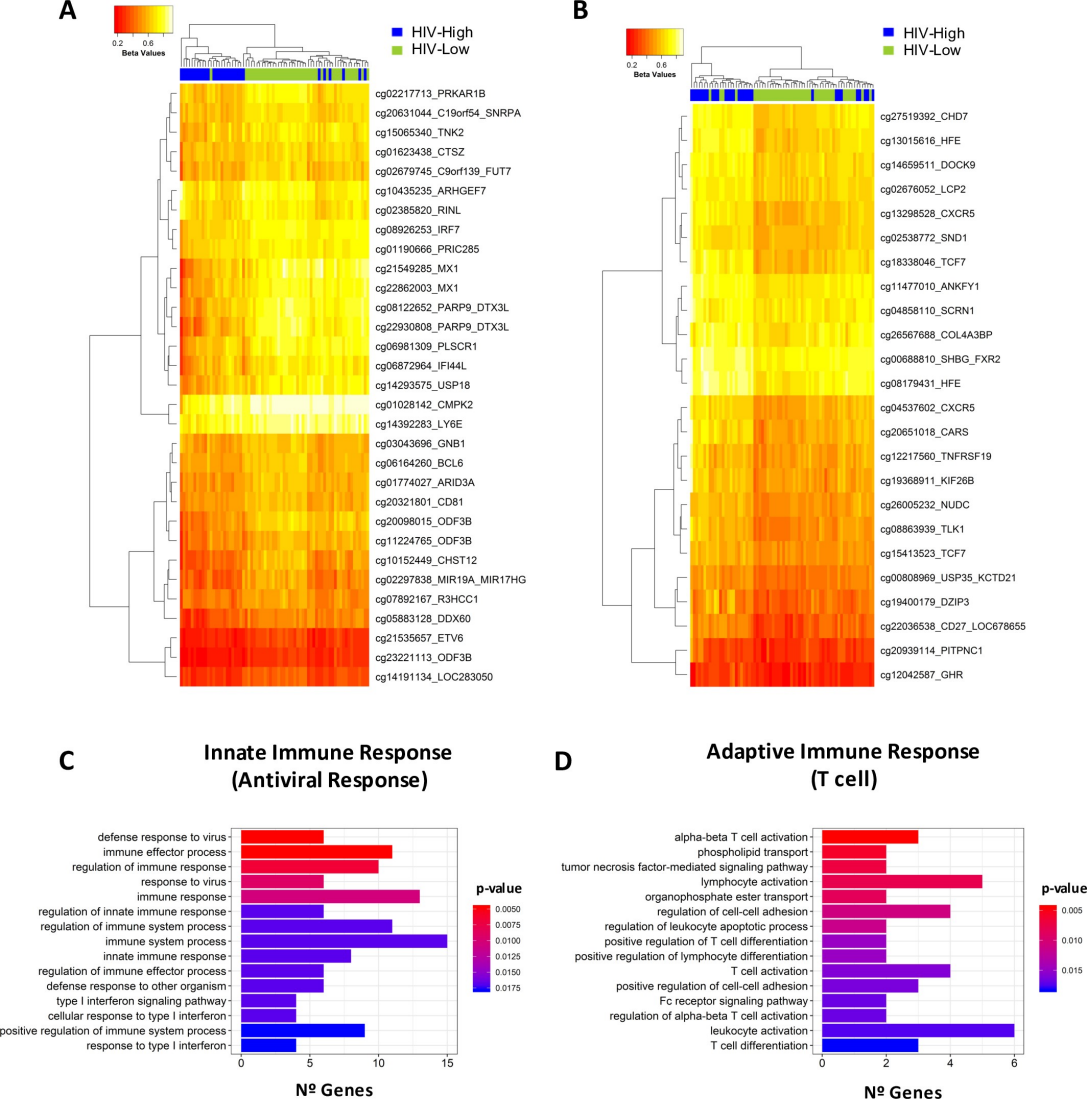
Relevant DMPs for classification in HIV-High and HIV-Low groups. Cluster analyses of the selected CpG positions for the classification of individuals (random forest model). Two clusters are showed in A and B as heatmaps that differentiate HIV-Low from HIV-High subjects. Distances are based on Manhattan and clustering, on complete linkage. Columns represent tested individuals, HIV-High (blue) and HIV-Low (green), and rows depict the Beta-values of the CpGs in each cluster. The colour scale from red to yellow represents from low to high methylation. (C-D) Histogram plots of the Gene Ontology Enrichment Analysis (clusterProfiler R/Bioconductor) for the two identified clusters (A and B) of DMPs. Pathways with the highest significance are shown; the complete output is in S3 Table.

The selected 55 DMPs clustered in two different groups that differentiate HIV-Low from HIV-High individuals (Table 1 and Fig 2A and 2B): Cluster 1 (Fig 2A) included 31 DMPs hypermethylated in HIV-Low individuals and cluster 2 (Fig 2B), included 24 DMPs hypomethylated in this same group. Gene ontology enrichment analysis (GEA) showed (Fig 2C and 2D, S3 Table) cluster 1 to be enriched for gene ontology (GO) categories involved in antiviral activity such as defense response to virus (GO:0051607, 6 genes), type I interferon signaling pathway (GO:0060337, 3 genes), or regulation of innate immune response (GO:0045088, 5 genes). In contrast, cluster 2 was represented by general immune activation GO categories including leukocyte activation (GO:0045321, 6 genes), T cell activation (GO:0042110, 4 genes), T cell differentiation (GO:0030217, 3 genes) or positive regulation of protein modification process (GO:0031401, 5 genes).

Overall, these data reinforce that the identified epigenetically regulated immune pathways of antiviral activity and innate immune activation are associated with HIV-1 pVL in the peripheral blood. Taken together, different levels of *in vivo* virus control are associated with markedly different methylation patterns of genes involved in antiviral innate and cellular adaptive host defense mechanisms.

### Differential methylation patterns are associated with proviral levels and markers of adaptive host immunity to HIV-1

To determine whether the DMPs associated with additional immune and virological markers beyond pVL, we correlated the methylation levels of the 55 DMPs identified above with proviral (total HIV-1 DNA) in PBMC lysates and extensive immune data, including measurements of cellular and humoral immunity to HIV-1 (S1 Table). T cell responses were determined by IFNg ELISpot assay against overlapping peptides of the entire consensus B HIV-1 proteome, and levels of neutralizing Ab (nAb) in plasma were determined against the HIV-1 BaL laboratory adapted strain. Table 2 shows the correlation data (Rho values, p<0.05, Spearman’s rank correlation test) between the relevant CpG positions contained in the two clusters and the different virological or immune parameters assessed.

**Table 2:** CpG positions correlation with virological and immunological parameters.

CpG	*Gene*	Cl	Freq. Random Forest	Viral Load (plasma HIV RNA copies/ml)		Proviral DNA		CD4 Counts		CTL Breadth		CTL Magnitude		nAb BaL	
				Rho	p-value	Rho	p-value	Rho	p-value	Rho	p-value	Rho	p-value	Rho	p-value
cg22930808	*PARP9* *DTX3L*	1	821	-0.73	9.39E-13	-0.59	2.20E-06	0.59	7.47E-08	0.36	4.09E-03	0.37	2.62E-03	-0.43	9.34E-04
cg20098015	*ODF3B*	1	724	-0.69	4.86E-11	-0.51	7.45E-05	0.53	2.36E-06	0.32	1.14E-02	0.34	6.17E-03	-	-
cg01028142	*CMPK2*	1	643	-0.61	1.53E-08	-0.46	4.79E-04	0.47	4.14E-05	-	-	0.29	2.00E-02	-	-
cg23221113	*ODF3B*	1	613	-0.74	3.87E-13	-0.56	8.55E-06	0.52	4.87E-06	0.37	2.63E-03	0.39	1.60E-03	-	-
cg10152449	*CHST12*	1	458	-0.68	7.47E-11	-0.54	2.56E-05	0.58	1.19E-07	0.44	2.92E-04	0.40	1.29E-03	-0.30	2.15E-02
cg02217713	*PRKAR1B*	1	452	-0.69	4.21E-11	-0.57	6.60E-06	0.60	3.88E-08	0.43	4.27E-04	0.33	8.85E-03	-0.26	4.66E-02
cg08122652	*PARP9* *DTX3L*	1	430	-0.69	4.61E-11	-0.53	3.35E-05	0.55	9.54E-07	0.29	2.30E-02	0.31	1.43E-02	-0.43	7.51E-04
cg14392283	*LY6E*	1	301	-0.71	4.64E-12	-0.60	1.14E-06	0.48	3.23E-05	0.26	3.71E-02	0.30	1.60E-02	-0.22	-
cg11224765	*ODF3B*	1	231	-0.66	3.45E-10	-0.56	9.95E-06	0.43	1.95E-04	0.31	1.29E-02	0.36	3.93E-03	-0.07	-
cg08926253	*IRF7*	1	225	-0.68	1.12E-10	-0.52	5.66E-05	0.51	8.22E-06	0.32	1.04E-02	0.31	1.49E-02	-0.30	2.27E-02
cg21535657	*ETV6*	1	168	-0.66	4.48E-10	-0.38	4.70E-03	0.45	8.15E-05	0.36	3.30E-03	0.30	1.52E-02	-	-
cg02679745	*C9orf139* *FUT7*	1	148	-0.58	1.46E-07	-0.47	2.64E-04	0.47	4.25E-05	0.37	2.77E-03	0.28	2.90E-02	-	-
cg01190666	*PRIC285*	1	126	-0.60	5.22E-08	-0.42	1.38E-03	0.48	2.22E-05	0.35	5.29E-03	0.32	1.15E-02	-	-
cg22862003	*MX1*	1	124	-0.55	8.10E-07	-0.44	6.93E-04	0.51	8.20E-06	0.37	2.58E-03	0.37	3.14E-03	-	-
cg06981309	*PLSCR1*	1	107	-0.61	2.10E-08	-0.51	5.90E-05	0.43	2.27E-04	0.26	4.10E-02	0.33	8.66E-03		
cg02297838	*MIR19A* *MIR17HG*	1	106	-0.64	2.00E-09	-0.52	4.05E-05	0.55	1.01E-06	0.37	2.55E-03	0.36	3.68E-03	-0.36	5.92E-03
cg01774027	*ARID3A*	1	95	-0.64	1.78E-09	-0.56	9.48E-06	0.61	1.95E-08	0.36	3.43E-03	0.33	7.40E-03	-	-
cg20631044	*C19orf54* *SNRPA*	1	95	-0.58	1.28E-07	-0.53	3.24E-05	0.55	7.25E-07	0.37	2.94E-03	0.27	3.15E-02	-	-
cg01623438	*CTSZ*	1	89	-0.53	2.01E-06	-0.40	2.58E-03	0.48	3.08E-05	0.36	3.81E-03	0.32	1.12E-02	-	-
cg14293575	*USP18*	1	78	-0.61	2.46E-08	-0.39	2.92E-03	0.41	3.73E-04	-	-	-	-	-	-
cg06164260	*BCL6*	1	76	-0.59	9.08E-08	-0.48	1.83E-04	0.51	7.27E-06	0.34	6.43E-03	0.26	4.00E-02	-0.31	1.94E-02
cg14191134	*LOC283050*	1	68	-0.61	1.81E-08	-0.46	4.82E-04	0.55	6.36E-07	0.36	3.76E-03	0.30	1.80E-02	-	-
cg21549285	*MX1*	1	67	-0.54	1.43E-06	-0.36	6.96E-03	0.49	2.03E-05	-	-	0.27	3.09E-02	-	-
cg15065340	*TNK2*	1	58	-0.52	4.35E-06	-0.33	1.39E-02	0.28	1.95E-02	-	-	-	-	-	-
cg05883128	*DDX60*	1	45	-0.64	2.92E-09	-0.44	7.95E-04	0.41	4.86E-04	0.26	3.68E-02	0.34	6.98E-03	-	-
cg03043696	*GNB1*	1	43	-0.44	1.21E-04	-0.36	6.44E-03	0.39	7.34E-04	-	-	-	-	-0.27	4.56E-02
cg10435235	*ARHGEF7*	1	42	-0.46	5.65E-05	-0.38	4.29E-03	0.46	7.08E-05	-	-	-	-	-	-
cg20321801	*CD81*	1	27	-0.55	8.37E-07	-0.48	1.90E-04	0.55	1.05E-06	0.32	1.03E-02	-	-	-	-
cg06872964	*IFI44L*	1	19	-0.50	1.31E-05	-0.37	5.13E-03	0.28	2.01E-02	-	-	-	-	-	-
cg07892167	*R3HCC1*	1	17	-0.53	2.45E-06	-0.31	2.09E-02	0.43	2.28E-04	0.28	2.88E-02	-	-	-	-
cg02385820	*RINL*	1	13	-0.41	4.10E-04	-0.32	1.79E-02	0.47	4.19E-05	-	-	-	-		
cg20939114	*PITPNC1*	2	607	0.52	3.62E-06	0.46	4.57E-04	-0.54	1.44E-06	-0.38	2.44E-03	-0.32	9.83E-03	-	-
cg02538772	*SND1*	2	374	0.59	6.34E-08	0.44	6.99E-04	-0.53	3.07E-06	-0.35	5.15E-03	-0.29	1.98E-02	-	-
cg15413523	*TCF7*	2	308	0.58	1.55E-07	0.39	3.60E-03	-0.37	1.47E-03	-0.37	3.19E-03	-0.39	1.69E-03	-	-
cg20651018	*CARS*	2	308	0.63	3.63E-09	0.54	2.05E-05	-0.54	1.38E-06	-0.42	7.09E-04	-0.35	4.75E-03	-	-
cg13015616	*HFE*	2	264	0.65	1.15E-09	0.52	4.18E-05	-0.55	7.32E-07	-0.43	5.12E-04	-0.34	5.96E-03	-	-
cg26005232	*NUDC*	2	240	0.60	4.86E-08	0.49	1.23E-04	-0.54	1.59E-06	-0.48	7.14E-05	-0.46	1.45E-04	-	-
cg08863939	*TLK1*	2	225	0.62	8.27E-09	0.61	9.40E-07	-0.63	4.96E-09	-0.47	8.41E-05	-0.43	5.14E-04	-	-
cg04537602	*CXCR5*	2	154	0.61	2.30E-08	0.56	7.38E-06	-0.63	5.23E-09	-0.43	4.13E-04	-0.35	4.79E-03	-	-
cg11477010	*ANKFY1*	2	141	0.63	7.14E-09	0.49	1.47E-04	-0.56	5.66E-07	-0.42	7.15E-04	-0.40	1.28E-03	-	-
cg18338046	*TCF7*	2	90	0.59	6.20E-08	0.53	2.75E-05	-0.51	5.44E-06	-0.39	1.39E-03	-0.31	1.47E-02	-	-
cg19400179	*DZIP3*	2	70	0.55	1.03E-06	0.38	4.47E-03	-0.43	1.97E-04	-0.25	4.40E-02	-	-	-	-
cg02676052	*LCP2*	2	69	0.44	1.44E-04	0.38	3.95E-03	-0.49	1.67E-05	-0.30	1.61E-02	-	-	-	-
cg00808969	*USP35* *KCTD21*	2	66	0.57	3.40E-07	0.40	2.82E-03	-0.57	2.04E-07	-0.32	1.17E-02	-0.28	2.43E-02	-	-
cg13298528	*CXCR5*	2	60	0.62	7.94E-09	0.50	8.60E-05	-0.57	2.03E-07	-0.42	6.33E-04	-0.31	1.27E-02	-	-
cg19368911	*KIF26B*	2	45	0.45	9.17E-05	0.32	1.91E-02	-0.48	2.94E-05	-0.37	3.10E-03	-0.34	6.26E-03	-	-
cg00688810	*SHBG* *FXR2*	2	43	0.56	4.15E-07	0.41	1.74E-03	-0.51	7.87E-06	-0.38	2.16E-03	-0.29	2.26E-02	-	-
cg27519392	*CHD7*	2	40	0.60	5.24E-08	0.42	1.43E-03	-0.57	3.09E-07	-0.44	2.91E-04	-0.37	2.57E-03	-	-
cg26567688	*COL4A3BP*	2	29	0.47	4.92E-05	0.36	6.53E-03	-0.55	7.26E-07	-0.38	2.34E-03	-0.34	5.98E-03	-	-
cg08179431	*HFE*	2	27	0.60	4.49E-08	0.51	7.53E-05	-0.54	1.65E-06	-0.38	2.32E-03	-0.28	2.46E-02	-	-
cg12042587	*GHR*	2	27	0.52	3.10E-06	0.48	2.43E-04	-0.38	1.05E-03	-	-	-0.27	3.46E-02	-	-
cg22036538	*CD27* *LOC678655*	2	27	0.53	2.54E-06	0.43	9.09E-04	-0.55	6.26E-07	-0.39	1.49E-03	-0.33	7.35E-03	-	-
cg04858110	*SCRN1*	2	24	0.49	1.87E-05	0.29	3.46E-02	-0.50	8.87E-06	-0.31	1.40E-02	-0.31	1.49E-02	-	-
cg12217560	*TNFRSF19*	2	21	0.46	7.12E-05	-	-	-0.56	5.11E-07	-0.33	7.89E-03	-0.26	4.06E-02	-	-
cg14659511	DOCK9	2	14	0.46	5.15E-05	0.41	2.10E-03	-0.52	3.28E-06	-0.34	5.92E-03	-	-	-	-

Rho is indicative of the Spearman’s rank correlation test. Only correlations with p-value <0.05 are shown.

Cl: Cluster.

Freq: Frequency

T cell Breadth: Number of reactive peptides measured by IFNg ELISpot assay.

T cell Magnitude: Median of SFC per 10^6^ PBMC resulted in IFNg ELISpot assay.

nAb BaL: Neutralizing antibodies to HIV-1-BaL laboratory adapted virus strain (1/IC50 of plasma)

DMPs in cluster 1, related to antiviral defense and innate immunity, showed the highest correlations with HIV-1 pVL and proviral levels. Specifically, the DMPs with the strongest correlations were the ones in the promoter regions of the *PARP9-DTXL3* (cg22930808 and cg08122652) and *ODF3B* genes (cg20098015, cg23221113 and cg11224765) that were also the most frequently selected in the random forest model (Table 2). The *PARP9* CpG positions were also correlated with the rest of variables measured, including levels of HIV-1 specific T-cells and nAb activity. Interestingly, in the same antiviral response group, *PARP9* (Fig 2B) clustered together with DMPs in *MX1* (cg22862003), *IRF7 (*cg08926253), *PLSCR1* (cg06981309), *USP18* (cg14293575) and *IFI44L* (cg06872964), all genes which were also correlated with HIV-1 pVL and proviral (Table 2). Additionally, the methylation levels of the CpG positions in *MX1*, *IRF7* and *PLSCR1* were related to the magnitude and breadth of the HIV-1-specific T-cell immune responses (Table 2). With the exception of *USP18*, all these genes were annotated in the functional category response to virus (GO:0009615) and immune effector processes (GO:0002252) among others (S3 Table). In addition, *USP18* was annotated together with *MX1*, *IRF7* and *PARP9* in the functional category of genes involved in the regulation of defense response (GO:0031347) and, with *MX1* and *IRF7*, in the category response to type I interferon (GO:0034340).

Similarly, in cluster 2, the DMP in the body of the *CARS* gene (cg20651018) was correlated with measured viral parameters and levels of HIV-1 specific T cell responses (Table 2). Furthermore, DMPs in promoter region of the *HFE* gene (cg13015616 and cg08179431) correlated with HIV-1 pVL, proviral levels, and breadth and magnitude of the virus-specific T cell responses. The *HFE* gene is involved in general T cell activation [16,17] and, interestingly, both DMPs showed the strongest correlation with other DMPs in the circle plot (S1 Fig). In addition, DMPs in the genes *CXCR5* (cg04537602 and cg13298528) and *TCF7* (cg15413523 cg18338046), both falling into the categories of lymphocyte and T-cell activation (GO:0046649 lymphocyte activation, GO:0046631 alpha-beta T-cell activation, GO:0042110 T-cell activation or GO:0030217 T-cell differentiation), were all correlated with the breadth and magnitude of the virus-specific T-cell response, as well as with pVL and proviral levels (Table 2). Overall, these correlation analyses highlight the potential relevance of epigenetic regulation of *PARP9*, *MX1*, *IRF7*, *USP18*, *PLSCR1* and *IFI44L* genes from cluster 1 (innate/antiviral response) and *HFE*, *CXCR5* and *TCF7* genes from cluster 2 (T-cell activation) in determining levels of virologic parameters, HIV-specific T cell responses and *in vivo* virus control.

### Methylation levels of candidate genes translate inversely into different mRNA levels

**Fig 3:**
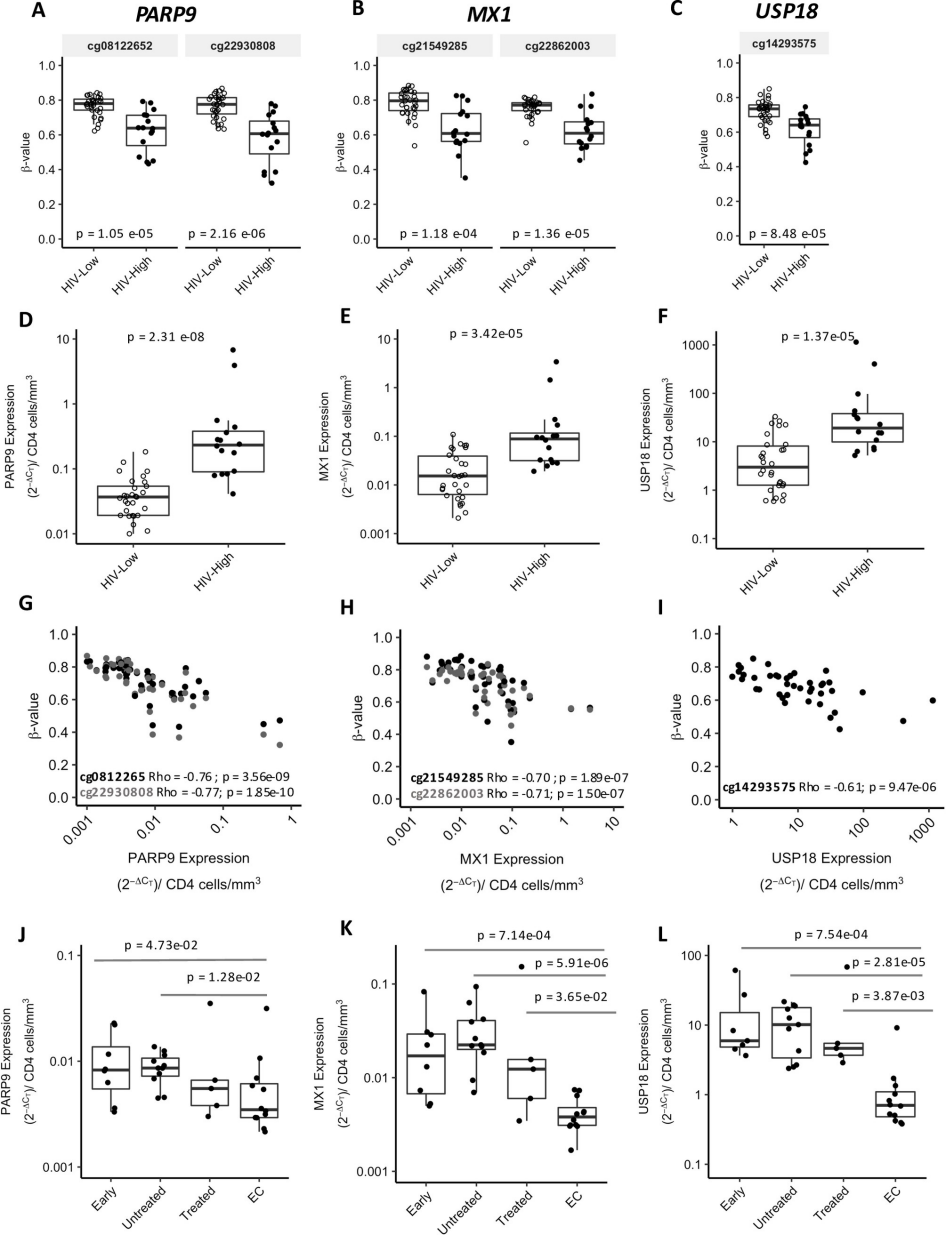
Epigenetic regulation confirmation of selected candidates and gene expression validation in unrelated cohorts. Boxplot for *PARP9* (A), *MX1* (B) and *USP18* (C) with the Beta-values in Y-axis and the HIV-High (n = 15) or HIV-Low (n = 31) groups in X-axis. (D-F) Boxplots of the transcriptional levels of *PARP9* (D), *MX1* (E) and *USP18* (F) analyzed by qRT-PCR, and represented here as the 2^-ΔCT^ method corrected by the number of CD4 counts in HIV-Low (n = 31) and HIV-High (n = 15). (G-I) Correlation plots, showing the inverse correlation between Beta-values of DMPs and the corresponding gene expression level *PARP9* (G), *MX1* (H) and *USP18* (I). (J-L) Boxplots indicating the gene expression level of PARP9 (J), MX1 (K) and USP18 (L) in the independent cohorts of HIV-1 infection: Early (Early infected untreated individuals, n = 8), Untreated (Chronic infected untreated individuals, n = 11), Treated (Chronic infected cART treated individuals, n = 5) and EC (Elite Controllers, n = 12). Mann Whitney test was applied for groups’ comparisons and Spearman’s rank correlation test, for correlation analysis. In all the cases, p-values <0.05 were considered significant.

To demonstrate a direct relationship between methylation levels and gene expression activity, we determined transcription levels for selected candidates. The top signals emerging from the random forest list (Table 1) included DMPs in promoters of genes *PARP9/DTX3L* and *ODF3B*, among others. Of importance, *PARP9/DTX3L* DMPs (cg22930808 and cg08122652) were strongly correlated with pVL and proviral (Table 2) and were included in the cluster enriched by antiviral gene ontology terms (Fig 2A and 2C). Focusing on that cluster 1, *PARP9* was in turn correlated with *MX1*, *PLSCR1*, *IFI44L* and *USP18*. Gene expression was assessed for *PARP9*, *MX1* and *USP18* using available total PBMC samples from the same individuals that were used for the initial methylome screening (n = 16 for HIV-High, n = 31 for HIV-Low, Table 1). HIV-Low individuals showed hypermethylation of *PARP9*, *MX1* and *USP18* (Fig 3A–3C); and indeed, all these three genes were less expressed in HIV-Low compared with HIV-High individuals (Fig 3D–3F). Thus, the observed DNA methylation patterns mediated epigenetic control of gene expression for these lead candidates (Fig 3G–3I). For the validation of the gene expression results and to confirm that the higher expression of these molecules was associated with uncontrolled HIV-1 infection, mRNA levels for *PARP9*, *MX1* and *USP18* were validated in independent cohorts of HIV-1 infected individuals. The cohorts included: Untreated early infected individuals (Early, n = 8), Chronic Untreated individuals (Untreated, n = 11), Chronic cART Treated individuals (Treated, n = 5) and Elite Controllers subjects (EC, n = 12) (S2 Table). The group of natural HIV-1 controllers showed consistently significant reductions in the expression levels of these genes compared with the rest of the studied groups (Fig 3J–3L).

**Fig 4:**
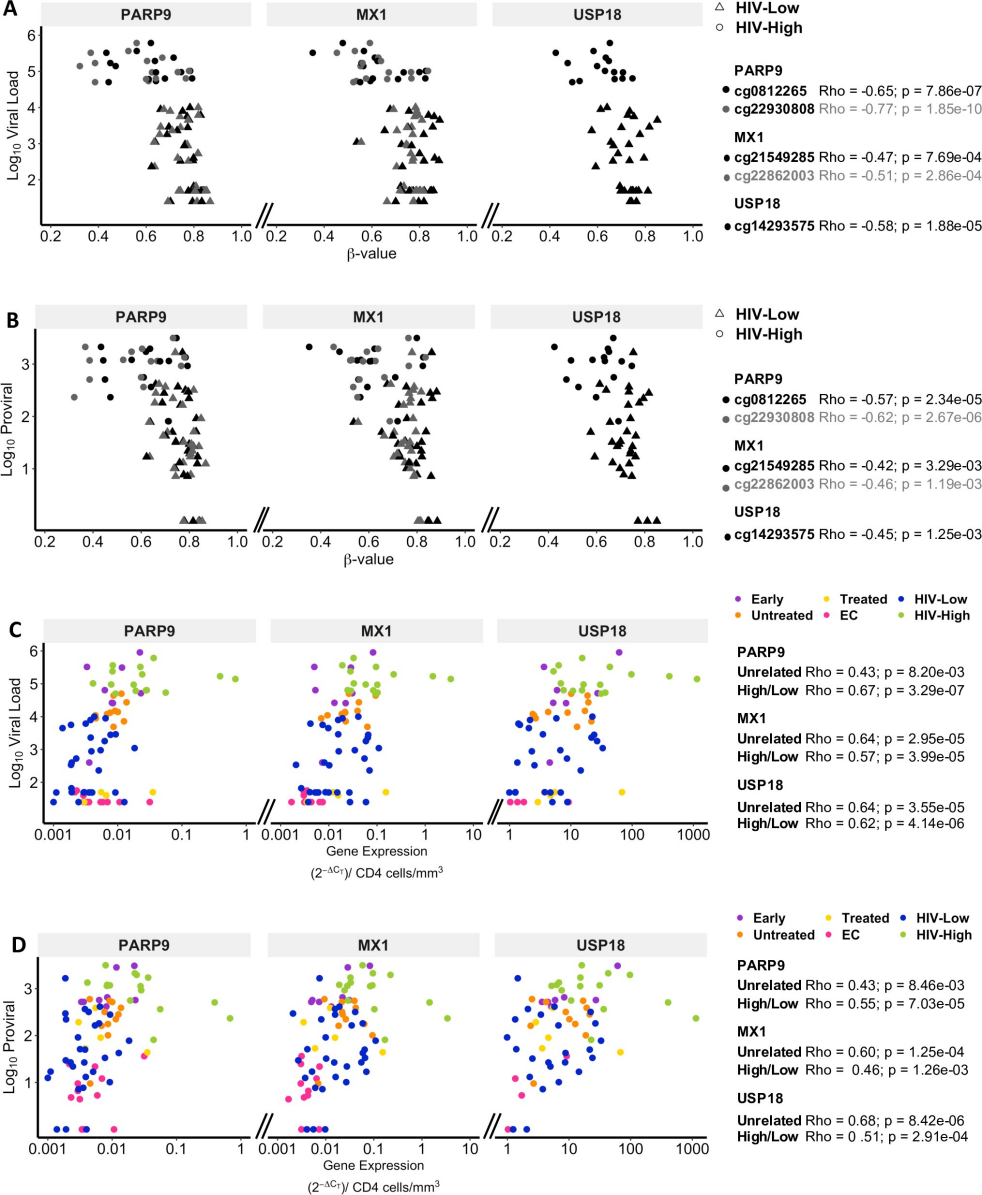
Correlations of gene expression and methylation levels of PARP9, MX1 and USP18 with HIV viral load and proviral. (A and B) PARP9, MX1 and USP18 correlation of methylation levels (X-axis) and HIV plasma viral load (A, Y-axis) or HIV proviral levels at PBMCs (B, Y-axis) in HIV-High and HIV-Low individuals. The different CpG positions are in different color (black and grey) and the two studied groups HIV-High and HIV-Low are indicated with a triangle and a square, respectively. (C and D) Correlation of PARP9, MX1 and USP18 gene expression determined by qRT-PCR and HIV plasma viral load (C, Y-axis) or HIV proviral levels at PBMCs (D, Y-axis). The different colors indicate the different groups of individuals including those in unrelated cohorts (Early in purple, Untreated in orange, Treated in yellow, EC in red) and HIV-High (green) and HIV-Low (blue). Spearman’s rank correlation test was applied for correlation analysis. In all cases, p-values <0.05 were considered significant.

When testing for potential correlations between levels of methylation, gene expression and viral parameters for all the individuals in the HIV-High and HIV-Low groups as well as from independent cohorts, the methylation and mRNA levels for all three assessed genes *PARP9*, *MX1* and *USP18*, strongly correlated with pVL and proviral levels (Fig 4). These data indicate that the differential expression levels of antiviral host factors (*PARP9*, *USP18* and *MX1)* between individuals with HIV-High and HIV-Low phenotypes are tightly related to epigenetic mechanism and could explain the different gene expression levels observed between HIV-1 elite controllers and the rest of HIV-1 infected subjects.

These conclusions were further supported by the re-analysis of an open access dataset (GSE53840, [18]). Using the same analysis approach as we did in our own data-set, this analysis identified the most relevant DMPs that discriminated patients with pVL<10.000copies/ml and pVL>10.000copies/ml, and further highlighted the relevance of PARP9 hypermethylation in HIV control (S4 Table).

### Opposite methylation patterns in interferon-related genes and T cell response genes are associated with HIV-1 control

**Fig 5:**
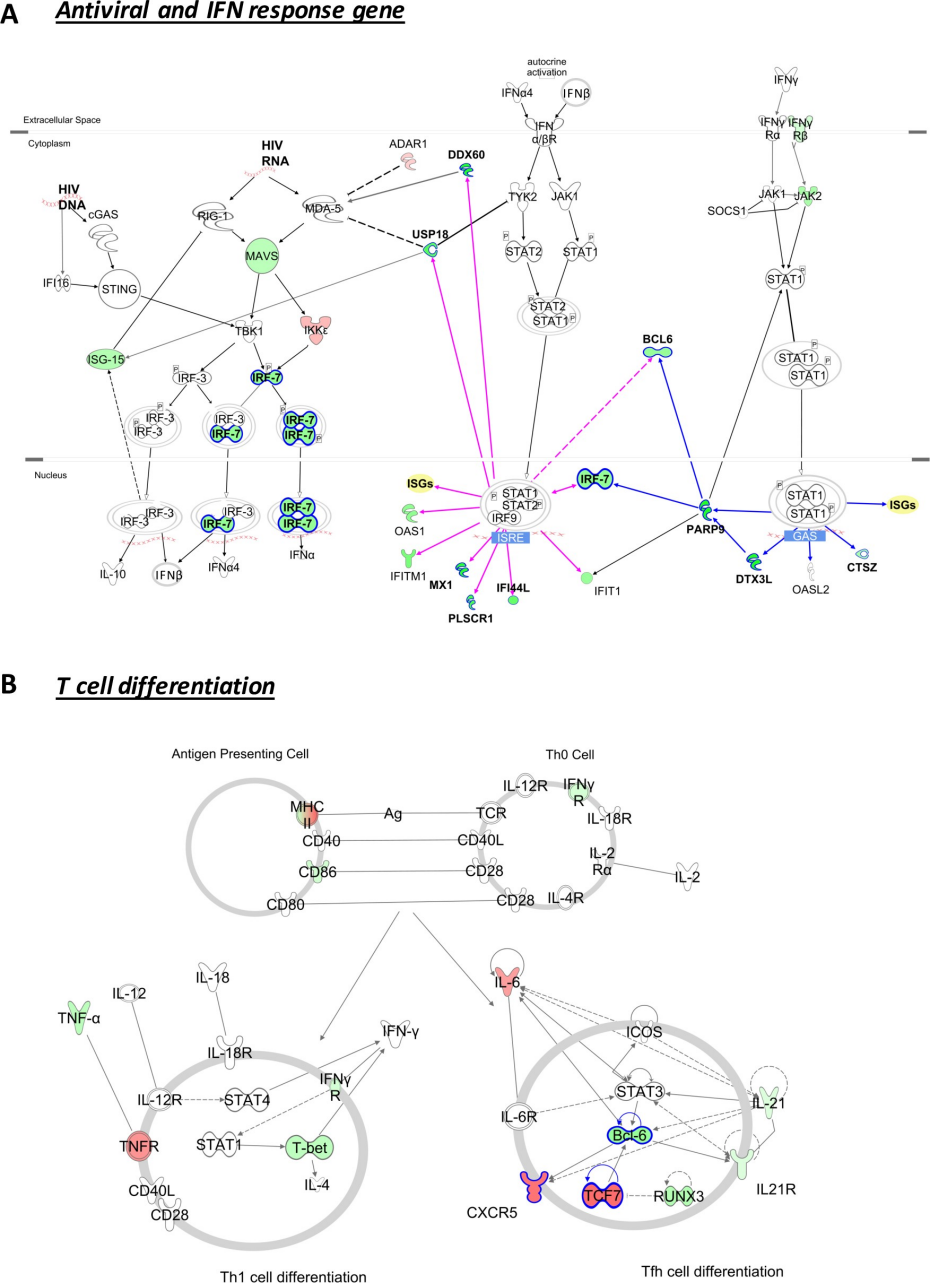
HIV-Low individuals show higher methylation levels in interferon stimulated genes and lower methylation levels in genes related to T follicular helper (Tfh). All differentially methylated CpG positions in the dataset (p<0.05) are shown in the context of two different pathways. A) Pathway representing genes associated with antiviral and interferon response focused on *Role of RIG-I like Receptors in Antiviral Innate Immunity*, *Antiviral activation of IRF by cytosolic pattern recognition receptors* and *Interferon Signaling* canonical pathways from IPA. B) Scheme showing *T Helper Cell Differentiation* canonical pathway from IPA combined with reported genes associated with Tfh phenotype. Coloring is based on methylation fold-change HIV-High/HIV-Low. Red refers to lower methylation in HIV-Low, and green higher methylation in HIV-Low. Also, pink and blue in (A) indicate the ISGs that are induced via type I or type II interferon, respectively. Molecules selected in the multivariate are highlighted with a blue border. Solid lines indicate direct interactions and dashed ones, indirect interactions.

To have a broader overview of the impact of the epigenetic dysregulation in the context of antiviral and T cell differentiation pathways, all the significant DMPs (p<0.05) in the dataset were studied. For the genes up-/downstream of the interferon signaling pathway (Fig 5A), we observed a higher methylation level in HIV-Low individuals, and the same trend was observed in genes of the antiviral pathway RIG-I/MDA-5 (*MAVS* and *IRF7*). On the other hand, HIV-Low individuals showed lower methylation levels and thus potentially higher protein levels for molecules involved in T-cell differentiation, including T follicular helper (Tfh) markers such as *CXCR5* and *TCF7* (Fig 5B).

Furthermore, in HIV-Low individuals we identified a negative correlation between the methylation levels of cg12459932 in *RUNX3* and the methylation levels of the CpG positions in *CXCR5* (cg04537602 Rho = -0.56, p- value = 3.67 x 10^−07^ and cg04537602 Rho = -0.58, p-value 1.07 x 10^−07^) and *TCF7* (cg15413523 Rho = -0.27, p-value = 0.02 and cg18338046 Rho = -0.47, p-value = 3.11 x 10^−05^). *RUNX3* (hypermethylation in HIV-Low) is involved in Tfh differentiation and has recently been reported to block CD8 effector T cell differentiation towards Tfh lineage [19]. As a consequence, in HIV-Low individuals *RUNX3* may be epigenetically repressed to favor the differentiation of Tfh cells. This epigenetic mechanism could explain the observed higher frequencies of this T-cell population in HIV-1 controllers [20].

## Discussion

The main objective of the present study was to investigate the importance of epigenetic regulation of host factors involved in the control of HIV-1 infection by assessing whether chronically untreated HIV-1 infected individuals with high or low plasma viral load levels show marked host genome DNA methylation patterns in the peripheral blood. Our data document significant differences in epigenetic signatures on a wide range of host proteins between these groups of patients and highlight the potential of methylome analyses to identify possible biomarkers associated with spontaneous control of HIV-1 infection. Therefore, the association of HIV-1 infection with differential DNA methylation patterns previously reported in studies comparing HIV+ and HIV- individuals [8,9], would extend the insights to markers and mechanisms underlying the spontaneous capacity to control HIV-1 replication in chronic infection.

In our study, two immune response Gene Ontology (GO) categories were found to be greatly under epigenetic regulation in untreated chronic HIV-1 disease with different virus control. While in HIV-Low individuals the innate immunity GO category (antiviral response and IFN inducible genes) was highly methylated, the GO category of adaptive immune response (T cell activation and differentiation) was poorly methylated compared to HIV-High individuals.

In particular, the most prominent DMPs signals detected in antiviral and interferon response GO categories and correlated with virological parameters included: *PARP9/DTX3L*, *MX1*, *USP18*, *IFI44L* and *PLSCR1*. These ISGs have been previously described to be involved in different viral infections, including HIV-1 [21–23]. Furthermore, the epigenetic regulation of the ISGs documented in our study, is in line with recently published data demonstrating a differential transcriptional regulation of ISGs (e.g *MX1*, *PARP* genes and *USP18* among others) in total PBMC of HIV normal progressors compared with HIV controller phenotypes [24]. Additional confirmation of the findings also stems from the gene expression patterns in the independent cohorts tested in our study, including treated and untreated HIV-1 infected individuals in different stages of HIV-1 infection including elite controller individuals.

The identified three genes play all a role in the interferon-mediated antiviral response: *PARP9* is increased *in vitro* models of HIV-1 infection [25,26] and, together with *DTX3L*, can target histone *H2BJ* and increase the expression of interferon stimulated genes (ISGs) [27,28]. Similarly, *USP18* has been described as a negative regulator of interferon response [21] and has been associated to HIV-1 replication as well [29]. Contrary to *MX2*, no role as HIV-1 restriction factor has been described for *MX1* [30,31]. However, three different studies associated the lower levels of *MX1* expression in the chronic phase of infection with relative virus control [24,32,33]. In line with these findings, our results further indicate that the epigenetic repression of these ISGs may contribute to achieving a spontaneous sustained control of HIV-1.

However, it needs to be stressed that our results identify factors and pathways involved in viral control based on methylome analyses in total PBMC. Thus, the identified factors do not necessarily need to have a direct impact on virus transcription in HIV-infected CD4 T cells. In fact, it may be quite likely that the majority of the identified host immune mechanisms that are associated with high or low viral load, and which are epigenetically controlled, originate from cells not infected by HIV (especially also considering the small number of infected cells in the peripheral blood). Interestingly, Morón-López et al (Morón-López et al. Abstract #228 CROI 2017), have reported full methylome data in isolated CD4 T cells from a small cohort of HIV infected patients with different levels of virus control. These results showed hypermethylation of USP18 in EC when compared to VC, supporting the results in our large cohort where hypermethylation of this gene was associated with better HIV control as well. Evidently, it will be very interesting to further define the methylome profile of this and other markers in latently or productively infected CD4 cells and other target cells of active replication and viral reservoir [34].

The role of activation of IFN pathways in the control of persistent HIV-1 infection is less clear, despite the strong signals observed in our analyses. On one hand, the activation of IFN genes in the acute phase of HIV-1 infection may benefit initial immune responses [35] and it has indeed been associated with protection from HIV-1 acquisition in a recent systems vaccinology study on the RV144 vaccine clinical trial [36]. On the other hand, the IFN response may become detrimental in the chronic phase of HIV-1 infection and has been related to poor antiviral response, higher virus loads and lower CD4 counts [37]. Supporting this observations, experiments in cART treated, HIV-1 infected humanized mice also showed that the blockade of IFNAR decreased immune activation and the size of the HIV-1 reservoir [38]. Similarly, Non-Human Primate (NHP) models using sooty mangabeys infected with Simian immunodeficiency virus (SIV) demonstrated an upregulation of *PARP9* and *USP18* among other ISGs that were later decreased in the chronic phase of SIV infection and, may be involved in the reduced disease progression in these animals despite the fact that they present with unusually high viral loads. In contrast, SIVmac239 infected Rhesus Macaques (as a model of continuing disease progression) maintain high levels of ISGs (including *MX1*) in the chronic phase. This findings derived from NHP models suggests that the downregulation of the IFN response that may help slow disease progression after SIV infection [39]. The same conclusion may be drawn from our dataset, where the higher methylation of ISGs and thus, lower expression levels, might improve the immune control of HIV-1 by downregulating IFN signaling and avoiding the systemic inflammation and consequent associated immune deterioration. Therefore, we suggest that the observed epigenetic brake on IFN signaling pathways may play a critical role in HIV-1 disease progression or HIV-1 control. Further analyses of individuals with known time since infection will be needed to prove this hypothesis. Furthermore, the study of individuals sampled before and after HIV-1 infection would also be insightful to determine when during the HIV-1 infection these differential DNA methylation patterns are established.

Individuals that initiate cART treatment within first days or weeks after HIV-1 show a reduced viral reservoir, but this is not enough to achieve an HIV-1 cure [40]. To date, many eradication and cure strategies aim to reactivate the latent virus with so-called latency reversing agents given in combination with therapeutic vaccines [41]. However, DNA methylation patterns existing in treated individual might well influence the outcome of such therapeutic interventions since our results show an epigenetic regulation of antiviral host factors and point to a possible epigenetic regulation of T cell differentiation. In regard to the latter, the two CpG positions identified in *CXCR5* and *TCF7* are of particular interest. While these genes have been associated with T follicular helper (Tfh) phenotypes in the past [42], with our study we now demonstrate that their expression is tightly controlled by epigenetic mechanisms and that this regulation may impact host defense and viral replication as both CpG positions were correlated with the breadth and magnitude of the HIV-1-specific T-cell responses, plasma viral load as well as the proviral levels. Furthermore, multiple DMPs were found in the Tfh pathway (Fig 5B) suggesting the epigenetic regulation of Tfh differentiation as a mechanism that might explain the higher frequencies of circulating Tfh in HIV-1 controllers [20]. As a consequence, the low methylation levels of these genes and elevated gene expression in HIV-Low individuals, may benefit effective anti-viral immune responses by preferentially driving T-cell differentiation towards a follicular-like phenotype, which in turn might avoid T-cell exhaustion of Th1-like cells.

Finally, aside from providing insights into natural control of HIV-1 infection, these findings may be of critical importance for the design and outcome of therapeutic interventions aimed at HIV-1 cure. Since DNA methylation marks are more stable than mechanisms regulating RNA expression [43], these methylation patterns may need to be restored in HIV-1 infected individuals with uncontrolled viral replication to attain sustained virus control. There are rapid advances in the field of gene editing that raise the hope that such modifications could indeed be implemented [43].

Of note, the obtained results were based on DNA methylation patterns on available dry PBMC cell pellets and further analysis of sorted cell type populations would be necessary to identify potential differences in cell-type specific DNA methylation patterns. At the same time, the study of total PBMC methylome allowed the identification of the most prominent DMPs associated with HIV control regardless of the cell subtype. Furthermore, potential bias associated with sample cell-type heterogeneity was partially overcome with a regression model adjusted for cell type proportion estimates [9,12] and CD4 correction. The latter helped to account for the significant differences in CD4 T cell counts in the two main comparison groups of HIV-Low and HIV-High. Importantly though, even without applying a CD4 counts correction, a strong selection of anti-viral and T cell response genes and the strong relevance of DMPs in PARP9 as well as USP18 are evident (S5 Table).

Despite these potential limitations, our study conducted in a well characterized HIV-1 infection cohort combined with comprehensive virological and immune functional assessments, provides clear evidence that differential epigenetic imprints on host DNA are tightly related to innate and adaptive immune responses against HIV-1 infection and relative control of *in vivo* viral replication. It will now be interesting to further our understanding of how DNA methylation itself is regulated in HIV-1 infected individuals and how this may allow to develop new therapeutic strategies to improve virus-specific immunity and HIV-1 cure. Furthermore, while the causal relationship between the epigenetic signals and virus control remains to be unraveled, our data show for first time that many host factors associated with immune control of HIV infection are under global epigenetic regulation, raising potential challenges for future cure strategies [11].

## Methods

### Ethics statements

The study was approved by the Comitè Ètic d’Investigació Clínica of Hospital Germans Trias i Pujol (CEIC: EO-12-042) and all participants provided written informed consent.

### Patients and samples

Chronic HIV-1 seropositive subjects (n = 70) were recruited at the Hospital Germans Trias i Pujol, Badalona, Spain, and the IMPACTA clinics in Lima, Peru. Subjects with plasma viral loads (pVL) <10,000 HIV-1 RNA copies/ml (range 25–9,999; median 2,760) in the absence of antiretroviral treatment were defined as "HIV-Low" (n = 41), while subjects with pVL >50,000 HIV-1 RNA copies/ml (range 50,295–1,200,000; median 237,459) were defined as "HIV-High" (n = 29) individuals (S1 Table). CD4 counts in the HIV-Low group ranged from 404–1,343 cells/mm3 (median 741) and from 11–726 cells/mm3 in the HIV-High group (median 283). Additional unrelated cohorts (S2 Table) were used for downstream validation of identified signals including: individuals sampled in early phase of HIV-1 infection (Early, median 3 months after seroconversion, n = 8, median viral load = 57,860), untreated individuals in chronic phase of infection (Untreated, n = 11, 1y before cART treatment, median viral load = 13,284), in chronic infected treated individuals (Treated, n = 5, 1y post cART initiation, undetectable viral load) and chronically infected elite controllers (EC, n = 13, undetectable pVL, at least 1y without treatment). For molecular assays, DNA and RNA was extracted using the QiaAmp Kit from available dry pellet PBMCs.

### Infinium Human Methylation450 Bead Chip

Bisulfite modification of 600 ng genomic DNA was carried out with the EZ DNA Methylation Kit (Zymo) following the manufacturer’s protocol. Next, 4 μL of bisulfite-converted DNA were used to hybridize on Infinium HumanMethylation450 BeadChip, following Illumina Infinium HD Methylation protocol. Chip analysis was performed using Illumina HiScan SQ fluorescent scanner and the intensities of the images were extracted using GenomeStudio (2010.3) Methylation module (1.8.5) software. Quality control, background correction and quantile normalization across arrays was performed using *Minfi* R/Bioconductor package [44] available for Bioconductor under the R statistical environment [45]. Methylation level (Beta-value) for each of the 485,577 CpG positions were calculated as the ratio of methylated signal divided by the sum of methylated and unmethylated signals plus 100. After a normalization step, probes related to X and Y chromosomes were removed. Function *dropLociWithSnps()* of *Minfi* R/Bioconductor package removed all the probes associated with SNPs in the body of the probe, specifically SNPs overlapping the CpG site and 1 or 2 bp extension from it (SBE, single base extension). Finally, the methylation scores were corrected for batch effect by using *comBat()* function from *sva* R/Bioconductor package [46]. Data is available at GEO under the accession number GSE140800.

### Definition of CpG methylation differences

A non-specific filtering was used to determine the most variable Beta-values for CpG positions according to standard deviation [47] and finally, we kept 56,513 CpG positions as the most variable CpG by selecting a threshold (sd > 0.47) for subsequent analysis. For the definition of CpG methylation differences, we applied an analysis based on an approach (CPACOR, [12]), previously used in DNA methylation studies in HIV-1 [9,48], which consists in a two-step regression model considering different confounders (age, sex, CD4 counts, PBMC composition and technical bias) (Fig 1A). A race confounder was not applied because all patients were of Caucasian origin. To correct for PBMC composition, the proportion of each cell type was estimated (WBCest) from the methylation intensity values of certain probes as described by Houseman et al. [49] and applied in function *estimateCellCounts()* in *Minfi* R/Bioconductor package [44]. For technical bias consideration (Fig 1A), a first model was applied using the 10 principal components (PCs) on 450K array control probes as covariates (PCs 1–10) in addition to the so-called above confounders:

Betacorrected∼WBCest+PCs1‐10+Age+Sex+CD4counts

Subsequently, a principal component analysis (PCA) was performed on the residuals of the technical bias correction model and 5 PCs (resPCs 1–5) were used in the second regression model:

Betacorrected∼Y+WBCest+PCs1‐10+Age+Sex+CD4counts+resPCs1‐5.

In this model (Fig 1A), Y refers to the individual class, HIV-High and HIV-Low. We selected those differential Beta-values (p < 0.05) between Y classes. The estimate of Y will retrieve the difference of methylation between the 2 groups considering that all the other covariates remain constant.

### Functional array analyses

Gene Ontology (GO) Enrichment Analysis was performed using the *ClusterProfiler* package from R/Bioconductor based on a hypergeometric test [50] in which the background were the 2649 gene-mapped DMPs. Heat-Maps (*gplots* R/CRAN pakage) and Hierarchical Clustering were performed with R software. For cluster analysis on selected CpG positions for classification of the two comparison groups, distances among DMPs were based on correlation values (Spearman’s Rho) and agglomeration, on average linkage method (R/CRAN package *Hmisc)*. The determination of the number of clusters was based on dendrogram height (h) and using the *fpc* R/CRAN package to determine the robustness of each cluster and determine the best agglomeration method. The analysis of molecule interactions was performed using Ingenuity Pathway Analysis Software IPA (QIAGEN Inc., https://www.qiagenbioinformatics.com/products/ingenuity-pathway-analysis) highlighting networks and canonical pathways affected by HIV-High and HIV-Low groups.

### Proviral (Total HIV-1-1 DNA) quantification

Proviral (Total HIV-1-1 DNA) was quantified in PBMC lysates by droplet digital polymerase chain reaction (ddPCR) in duplicate, as described previously [51]. Briefly, two different primers/probe sets annealing to the 5′LTR and Gag regions, respectively, were used to circumvent sequence mismatch in the patients’ proviruses, and the *RPP30* housekeeping gene was quantified in parallel to normalize sample input. Raw ddPCR data were analyzed using the QX100 Droplet Reader and the QuantaSoft v.1.6 software (Bio-Rad).

### Measurements of adaptive host immune responses to HIV-1

T cell immunity to HIV-1 was assessed in cryopreserved isolated PBMC by IFNg ELISpot assay (1x10^5^ PBMC/well), using a set of 410 overlapping peptides (OLPs, (18mers overlapping by 15 aa) covering the consensus B HIV-1 viral proteome as described elsewhere [52]. The breadth (number of reactive OLP) and magnitude (spot forming cells (SFC) per 10^6^ PBMC) were recorded. Neutralization activity of plasma samples was evaluated using a TZM-bl neutralization assay as described elsewhere [53,54]. Briefly, five-fold serial dilutions of plasma samples (inactivated for 1 hour at 56°C) were incubated in 96-well plates in duplicates for 1 hour with 200 TCID50 of the laboratory adapted viral strain HIV-1-BaL, Vesicular stomatitis virus (VSV) envelope pseudotyped HIV-1 was included as a control for HIV-1 unspecific neutralization. Then, 10^4^ TZM-bl cells were added per well and incubated for 48 hours at 37°C and 5% CO_2_. Luciferase activity was determined by luminometry using the BriteLite plus reagent (PerkinElmer) and the Fluoroskan Ascent FL luminometer (Labsystem). The percentage of neutralization was calculated as: % Neutralization = [1−(R−Rcc/Rvc)] ×100; where R = RLU (relative light units) of the tested plasma, Rcc = RLU of cell alone and Rvc = RLU of virus alone. The IC50 (dose inducing 50% of total inhibitory capacity) was calculated and results are shown as reciprocal dilution.

### Real Time PCR

Dry cell pellets were conserved in RNAprotect Cell Reagent (Qiagen) and used for RNA extraction (RNeasy Plus Mini Kit, Qiagen) and retro-transcription (SuperScriptIII First-Strand Synthesis SuperMix). The cDNA was used for RT-PCR using Taqman Gene Expression Assays for detection of: *PARP9* (Hs00967084_m1), *MX1*(Hs00895608_m1), *USP18* (Hs00276441_m1) and Tata binding Box protein (TBP) (Hs99999910_m1) as housekeeping gene with 7500 Fast Dx Real-Time PCR Instrument (Applied Biosystems). The relative expression was calculated as Relative Expression = 2^-ΔCT^ (CT = the median of crossing thresholds from 3 replicates).

### Statistics

To identify robust methylation profiles in extreme outcomes of HIV control, we decided for our initial analysis, to classify individuals in our main cohort into two comparison groups (HIV-High and HIV-Low) rather than conducting correlation analysis with viral load as a continuous variable. To this end, we applied a random forest analysis to determine the most relevant features considering the contribution of all the CpGs at the same time. This is also a suitable approach to deal with data collinearity [55]. Accordingly, we applied a random forest classification model based on the previously identified 2649 gene annotated DMPs with five-fold cross validation in an iterative way (1000 iterations) with the *CMA* R/Bioconductor [15].

The reliability of the model to classify individuals was assessed with the area under the curve (AUC). The output of this analysis provides a ranking of DMPs (frequency) according to the number of times that each CpG position was selected in the random forest analyses (AUC = 0.92). We kept those CpG sites that were selected for a minimum of 10 times. Spearman’s rank correlation test was used for correlations among DMPs and clinical, virological and immunological parameters including: CD4 counts, plasma viral load, Proviral levels, T cell responses (T cell breadth and magnitude) and plasma neutralizing antibodies (nAb) activity against the virus strains BaL (see corresponding methods section). The correlation data among DMPs were plotted using *Circlize* R/CRAN package [56]. Finally, univariate statistical analyses for group comparisons were based on non-parametric Mann-Whitney test and p-value < 0.05 was considered statistically significant.

## Supporting information

S1 Fig Random Forest selected CpG sites correlate among them and with antiviral and immunological parameters. Circos plot showing DMPs associated with each of the viral and immunological parameters and their interrelation. The significant correlations (p-value < 0.05) with CpG sites and a maximum of 15 CpG sites is shown per each parameter (based on higher Spearman’s Rho values): viral load (orange), HIV-proviral (yellow), breadth (purple) and magnitude (green) of the virus specific T cell response and neutralizing antibody capabilities against NL43 (pink) and BaL (grey). The inter-relation between the different CpG sites methylation levels (only correlations with Spearman’s Rho > |0.6| and p-value < 0.05) are also shown (In green there are the positive correlations, and in red, the negative ones).
(TIF)

S1 Table Clinical information of HIV-infected individuals ND: Not determined. T cell Breatdh: Number of reactive peptides. T cell Magnitude: Median of SFC per 10^6^ PBMC. nAb NL43: Neutralizing antibodies to NL43 (1/IC50 of plasma). nAb BaL: Neutralizing antibodies to NL43 (1/IC50 of plasma). M: Male. F: Female.
(XLSX)

S2 Table Clinical information independent cohorts Clinical information of independent cohorts including age, sex, viral load and CD4 counts. M: Male F: Female
(XLSX)

S3 Table Gene Enrichment Analysis S3 Table contain 2 tables (S3 Table cluster 1 and S3 Table cluster 2) with the results from the gene enrichment analysis performed using clusterProfiler R/Bioconductor for each of the identified clusters. (XLSX)

S4 Table Classificatory CpG positions into the groups of HIV-High or HIV-Low for validation dataset GSE53840. p-value: p-value of the regression model applied to determine DMPs. CpG positions are ordered according the frequency of selection by random forest model. HIV-High = pVL > 10.000copies/ml. HIV-Low = pVL < 10.000copies/ml. Chr = Chromosome.
(XLSX)

S5 Table Classificatory CpG positions into the groups of HIV-High or HIV-Low without CD4 counts correction (study dataset: GSE140800). p-value: Makes reference to the p-value of the regression model (without CD4 counts correction) applied to determine DMPs. CpG positions are ordered according the frequency of selection by random forest model. HIV-High = pVL > 50.000copies/ml. HIV-Low = pVL < 10.000copies/ml. Chr = Chromosome.
(XLSX)

S1 Data Excel spreadsheet with data for the different figures. Fig 1B, Fig 1C, Fig 2A, Fig 2B, Fig 2C, Fig 2D, Fig 3A–3I, Fig 3J–3L, Fig 4A and 4B, Fig 4C and 4D and Fig 5.
(XLSX)
