# Supplementary figures and images for: Methylation regulation of Antiviral host factors, Interferon Stimulated Genes (ISGs) and T-cell responses associated with natural HIV control
Source: PLoS Pathog. 2020 Aug 6;16(8):e1008678. doi: 10.1371/journal.ppat.1008678 (PMC7410168; doi:10.1371/journal.ppat.1008678)

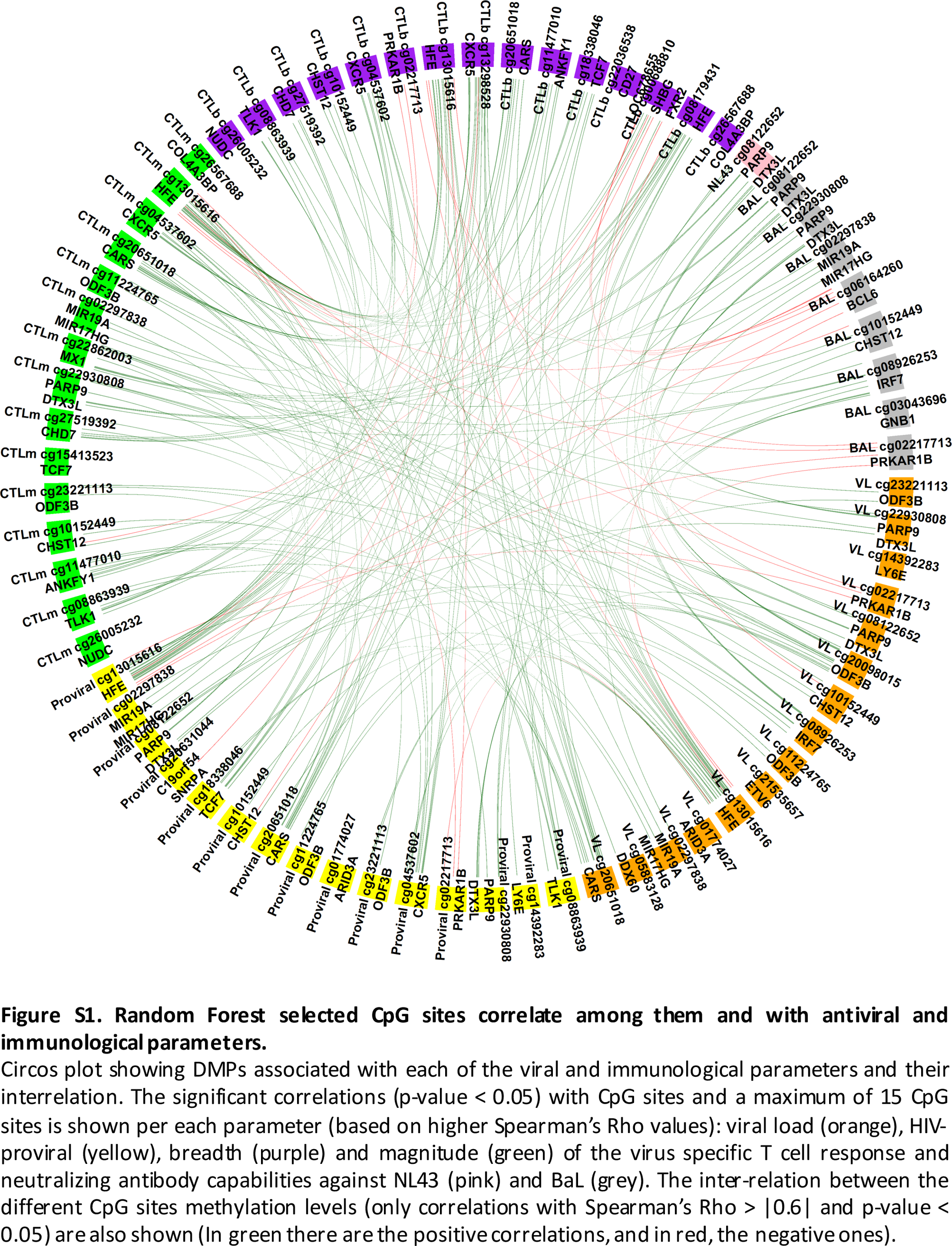

Supplement: S1 Fig — Circos plot showing DMPs associated with each of the viral and immunological parameters and their interrelation. The significant correlations (p-value < 0.05) with CpG sites and a maximum of 15 CpG sites is shown per each parameter (based on higher Spearman’s Rho values): viral load (orange), HIV-proviral (yellow), breadth (purple) and magnitude (green) of the virus specific T cell response and neutralizing antibody capabilities against NL43 (pink) and BaL (grey). The inter-relation between the different CpG sites methylation levels (only correlations with Spearman’s Rho > |0.6| and p-value < 0.05) are also shown (In green there are the positive correlations, and in red, the negative ones). (TIF) [file ppat.1008678.s001.tif]
